# Active Case Finding (ACF) of Tuberculosis Among School Students: Insights From a Tribal District in Seoni, Madhya Pradesh, India

**DOI:** 10.7759/cureus.86711

**Published:** 2025-06-25

**Authors:** Yogesh Sharma, Manohar Bhatia, Vikas Dwivedi, Bikramjeet Mitra, Vikas Sabharwal, Varsha Rai

**Affiliations:** 1 Community Medicine, World Health Organization Regional Office, Bhopal, IND; 2 Community Medicine, Gajra Raja Medical College (GRMC), Gwalior, IND; 3 Community Medicine, Government Medical College, Datia, Datia, IND; 4 Community Medicine, Chhindwara Institute of Medical Sciences, Chhindwara, IND; 5 Preventive Medicine, World Health Organization, Gwalior, IND; 6 Tuberculosis, State TB Office, Bhopal, IND

**Keywords:** active case finding, community-based intervention, health promotion, school-based screening, tribal health

## Abstract

Background: Tuberculosis (TB) remains a major public health concern in India, particularly among tribal populations who face structural and socioeconomic barriers to healthcare access. Despite ongoing efforts by the National Tuberculosis Elimination Program (NTEP), many tribal districts report persistently high TB burdens. Active case finding (ACF) has demonstrated effectiveness in improving early TB detection and reducing transmission.

Objective: This study aims to assess the effectiveness of a school-based ACF initiative in identifying symptomatic individuals and potential TB cases within the tribal communities of Seoni district, Madhya Pradesh.

Materials and methods: A cross-sectional record review was conducted from March to April 2024 across nine educational institutions in Seoni district. School students, oriented by NTEP staff, screened their family members using a 12-question format that addresses TB symptoms and risk factors. Data from 2,210 individuals were compiled and analyzed using Jamovi version 2.3.28 (Computer Software; retrieved from https://www.jamovi.org).

Results: Thirty percent with appetite loss and 26% with >5 kg weight loss underwent TB testing, with these symptoms showing strong associations with testing (p < 0.001). Only four individuals were diagnosed with TB. History of TB in the past one to two years and generalized weakness were significant predictors of diagnosis. Logistic regression revealed age, symptom count, and TB history as significant predictors of testing and diagnosis.

Conclusion: School-based ACF is a feasible and promising strategy for TB detection in tribal areas. While the diagnostic yield was low, the model showed potential for broader implementation and community mobilization. Further studies are warranted to evaluate the long-term impact and optimize the implementation.

## Introduction

Tuberculosis (TB) remains a significant public health issue in India, particularly among tribal communities, who face socioeconomic and geographical barriers to healthcare access [1]. Despite the efforts of the National Tuberculosis Elimination Program (NTEP), tribal districts consistently report a high TB burden, partly due to diagnostic delays and limited health-seeking behavior [2]. In these settings, innovative active case-finding (ACF) strategies are essential for early detection and prompt treatment.

Community-based ACF has proven effective in tribal populations by overcoming obstacles such as limited awareness, stigma, and challenges in accessing healthcare [3]. Research shows that systematic screening of high-risk groups, including tribal communities, can greatly improve TB detection rates, enhance treatment outcomes, and reduce transmission [4]. Outreach programs with trained community health workers (CHWs) have successfully increased TB notifications and reduced pretreatment loss to follow-up [5].

A novel approach involves engaging school students as active participants in ACF, particularly in tribal districts like Seoni, Madhya Pradesh. Schools serve as vital community centers, and students can act as effective channels for health awareness, helping to identify symptomatic individuals within their families and communities. School students are selected as active participants in ACF in tribal districts due to their accessibility, community trust, ability to reach households, cost-effectiveness, and potential to spread awareness and reduce TB stigma within their families and communities [6]. The recent evidence from high TB burden tribal communities shows that community-based interventions significantly reduce TB prevalence by improving early detection and treatment uptake [7].

Implementing school-based ACF has the potential to bridge gaps in healthcare access, empower school students as health ambassadors in tribal districts, and contribute to India's ambitious goal of eliminating TB by 2025, five years ahead of the Sustainable Development Goals. By integrating education with public health initiatives, this approach can ensure a sustainable, community-driven model for TB control in rural, underserved regions. The study aims to assess the feasibility and utility of a school-based ACF initiative for identifying individuals with TB symptoms and potential TB cases in the tribal communities of Seoni district, Madhya Pradesh.

## Materials and methods

Study setting and population

This is a community-based, cross-sectional, observational study conducted in nine government educational institutions in the Seoni district of Madhya Pradesh over two months, from March to April 2024. The study population comprised school students from classes 9-12, aged 14-18 years, who voluntarily participated in a TB screening activity as part of a school-based ACF initiative. In addition, secondary data from the ACF module of the Ni-Kshay software (Central TB Division, India), corresponding to the same timeframe, were incorporated into the study to supplement and cross-validate the findings.

Study design

A cross-sectional observational design, utilizing a questionnaire-based survey, was employed. The study involved a review of TB screening forms and verification of corresponding Ni-Kshay data.

Sampling method and sample size

A universal sampling approach was employed, in which all eligible students from the selected nine schools were invited to participate. A total of 437 students from classes 9 to 12 participated, providing a total of 2,210 completed screening forms, which included both self-screening and screening of household members. No formal sample size calculation was performed, as the study utilized all available programmatic data from the participating schools.

Inclusion criteria

Students enrolled in classes 9-12 (aged 14-18 years) in participating schools who provided written assent and returned the completed TB screening form within the data collection window were included.

Exclusion criteria

Students absent on the day of form distribution, or those who did not return the form or declined to participate, were excluded.

Procedure and data collection

Students were selected as facilitators for household-level TB symptom screening following a structured sensitization session conducted by the NTEP team. Each student received a validated, standardized TB screening questionnaire in the local language (Hindi), consisting of 12 questions covering TB symptoms, diabetes, tobacco use, and alcohol consumption. These forms served as both health education tools and primary screening instruments. Students completed the forms for themselves and also screened household members. Each student was given three days to return the completed forms.

**Figure 1:**
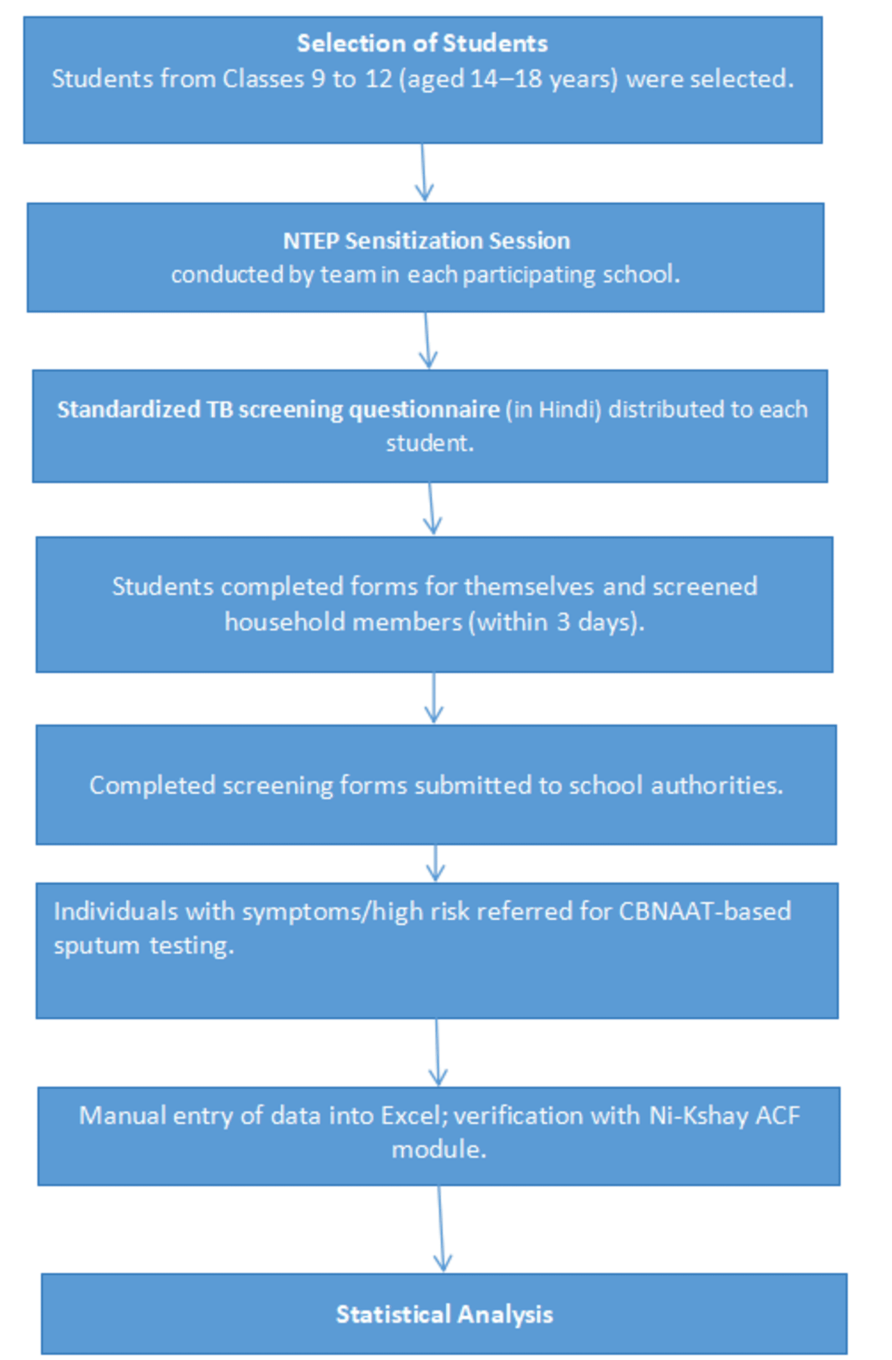
Flowchart showing process of data collection NTEP: National Tuberculosis Elimination Program; CBNAAT: cartridge-based nucleic acid amplification test; ACF: active case finding

A total of 2,210 completed screening forms were collected from 437 students across the nine schools. The data were manually entered and cross-referenced with the Ni-Kshay ACF module for validation and outcome tracking. Figure 1 shows the whole process.

Data analysis

Data were compiled into Microsoft Excel (Microsoft Corporation, Redmond, WA) and analyzed using descriptive statistics. The prevalence of TB symptoms was calculated, and the distribution of associated risk factors (diabetes, tobacco, and alcohol use) was evaluated. Individuals flagged as high risk were referred for cartridge-based nucleic acid amplification test (CBNAAT)-based sputum testing, and results were relayed back to the district TB program.

Ethical considerations

Ethical approval was obtained from the Institutional Ethics Review Committee, Bundelkhand Medical College, Shahdol. Administrative clearance was secured from the State TB Office, Bhopal, and the District TB Officer, Seoni. Written informed consent was obtained from all participating students. For minors, written parental consent was obtained through the students, who were instructed to explain the process at home before screening their family members. All data were anonymized to ensure confidentiality.

## Results

**Figure 2:**
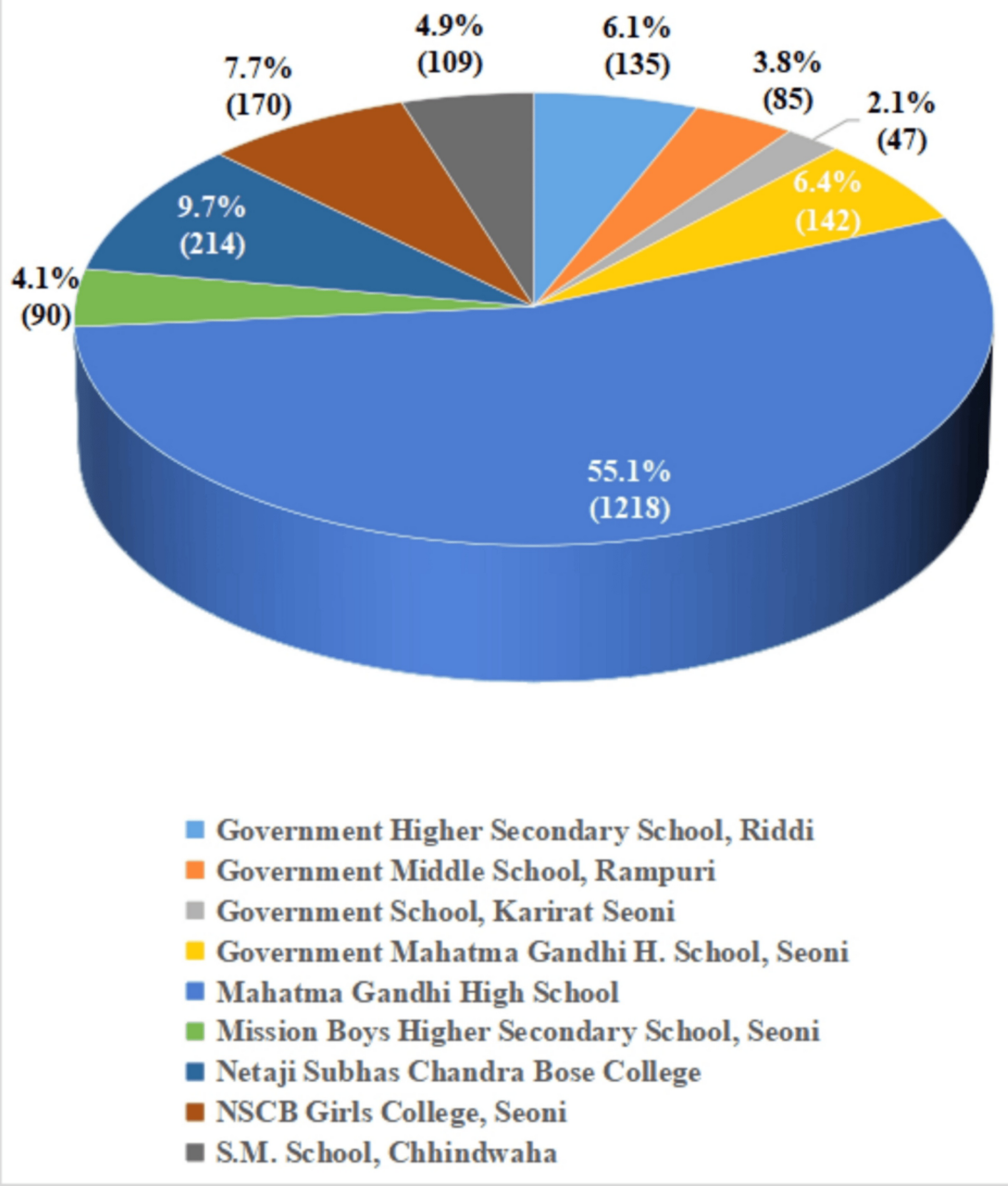
Distribution of family members screened by ACF team in different schools ACF: active case finding

A total of 2,210 family members were screened for TB across nine educational institutions. The mean age of participants was 29.2 ± 16.2 years. The highest proportion of individuals screened was from Mahatma Gandhi High School, accounting for 55.1% (n = 1,218) of the total. Other notable contributions came from Netaji Subhas Chandra Bose College (9.7%, n = 214), Netaji Subhash Chandra Bose Girls College, Seoni (7.7%, n = 170), and Government Mahatma Gandhi Higher Secondary School, Seoni (6.4%, n = 142). Smaller proportions were reported from Government Higher Secondary School, Riddi (6.1%, n = 135), Mission Boys Higher Secondary School, Seoni (4.1%, n = 90), S.M. School, Chhindwara (4.9%, n = 109), Government Middle School, Rampuri (3.8%, n = 85), and Government School, Karirat, Seoni (2.1%, n = 47) (Figure 2).

Among participants reporting appetite loss and weight loss >5 kg in the past month, 30.4% and 26.1%, respectively, underwent TB testing, both showing strong and statistically significant associations (χ² = 161 and 215; p < 0.001). Generalized weakness was also significantly associated with TB testing (26.1%, χ² = 52; p < 0.001). Symptoms such as cough >14 days (13.0%) and fever >14 days (4.3%) showed significant associations with testing (χ² = 99.2 and 24.2; p < 0.001). Chest pain in the last month (4.3%, p = 0.013) and pain in the neck/axilla (2.2%, p = 0.012) were also significantly associated with testing, though with smaller effect sizes. Night sweats approached statistical significance (p = 0.051), while history of TB in the last one to two years showed a significant association with testing (χ² = 14.4; p < 0.001). The presence of blood in sputum, however, was not significantly associated with testing (p = 0.837) (Table 1).

**Table 1:** Association between TB symptoms and TB testing among surveyed individuals (n = 2,210) ^*^p < 0.05; ^**^p < 0.001; NS: not significant Fischer's exact test was used TB: tuberculosis

Symptoms	TB testing done, n (%)	TB testing not done, n (%)	χ² value	p value
Fever >14 days	2 (4.3%)	44 (95.7%)	24.2	<0.001^**^
Cough >14 days	6 (13.0%)	40 (87.0%)	99.2	<0.001^**^
Weight loss >5 kg in the last month	12 (26.1%)	34 (73.9%)	215	<0.001^**^
Appetite loss	14 (30.4%)	32 (69.6%)	161	<0.001^**^
Chest pain since last month	2 (4.3%)	44 (95.7%)	6.21	0.013^*^
Blood in sputum (in the last six months)	0 (0%)	46 (100%)	0.04	0.837^NS^
Night sweats	2 (4.3%)	44 (95.7%)	3.8	0.051^NS^
Generalized weakness	12 (26.1%)	34 (73.9%)	52	<0.001^**^
Pain in the neck/axilla	1 (2.2%)	45 (97.8%)	6.28	0.012^*^
History of TB in the last 1-2 years	1 (2.2%)	45 (97.8%)	14.4	<0.001^**^

Of the total population surveyed, only four individuals were diagnosed with TB. Among reported symptoms, a significant association with TB diagnosis was observed for generalized weakness (p = 0.046), with one of the 98 individuals reporting weakness testing positive for TB. A strong and highly significant association was also found with a history of TB in the past one to two years (χ² = 183, p < 0.001), indicating recent TB history as a critical risk factor. Other symptoms, including fever >14 days, cough >14 days, >5 kg weight loss, appetite loss, chest pain, hemoptysis, night sweats, and pain in the neck/axilla, did not show statistically significant associations with TB diagnosis (all p > 0.05) (Table 2).

**Table 2:** Association between TB symptoms and TB diagnosis among surveyed individuals (n = 2,210) ^*^p < 0.05; ^**^p < 0.001; NS: not significant Fischer's exact test was used TB: tuberculosis

Symptom/screening	TB diagnosis done, n (%)	TB diagnosis not done, n (%)	χ²	p value
Fever >14 days	0 (0%)	7 (100%)	0.013	0.91^NS^
Cough >14 days	0 (0%)	16 (100%)	0.029	0.864^NS^
Weight loss >5 kg in the last month	0 (0%)	30 (100%)	0.055	0.814^NS^
Appetite loss	0 (0%)	52 (100%)	0.097	0.756^NS^
Chest pain since last month	0 (0%)	20 (100%)	0.037	0.848^NS^
Blood in sputum (in the last six months)	0 (0%)	2 (100%)	0.004	0.952^NS^
Night sweats	0 (0%)	27 (100%)	0.05	0.824^NS^
Generalized weakness	1 (1%)	97 (99%)	4	0.046^*^
Pain in the neck/axilla	0 (0%)	6 (100%)	0.011	0.917^NS^
History of TB in the last one to two years	1 (33.3%)	2 (66.7%)	183	<0.001^**^

A binomial logistic regression model was used to identify predictors of undergoing TB testing, which showed a good fit (deviance = 271, Akaike information criterion = 285, McFadden’s R² = 0.394). Significant predictors included age (β = 0.0579, p < 0.001; odds ratio, OR = 1.06, 95% CI: 1.04-1.08), number of symptoms (β = 1.8628, p < 0.001; OR = 6.44, 95% CI: 4.14-10.02), and history of TB in the last one to two years (β = 3.0714, p = 0.041; OR = 21.57, 95% CI: 1.13-412.69). History of diabetes and tobacco/alcohol use were not significant predictors (p > 0.98) (Table 3).

**Table 3:** Multivariate logistic regression analysis of predictors associated with TB testing among individuals ^*^p < 0.05; ^**^p < 0.001; NS: not significant Multivariate logistic regression analysis was used The intercept indicates baseline log odds when all predictors are set to zero β: regression coefficient; SE: standard error; Z: Z-statistic; OR: odds ratio; CI: confidence interval; TB: tuberculosis

Predictor	Estimate (β)	SE	Z	p value	OR	95% CI for OR
Intercept	-17.37	2,399.54	-0.0072	0.994^NS^	2.84 × 10^-8^	0-∞
Age of family member (years)	0.0579	0.011	5.254	<0.001^**^	1.06	1.04-1.08
Number of symptoms	1.8628	0.2253	8.2687	<0.001^**^	6.44	4.14-10.02
History of TB in the last 1-2 years (yes vs. no)	3.0714	1.5058	2.0397	0.041^*^	21.57	1.13-412.69
History of diabetes (yes vs. no)	-13.7924	768.89	-0.0179	0.986^NS^	-	-
Tobacco/alcohol use (yes vs. no)	12.895	2,399.5445	0.0054	0.996^NS^	-	-

Another binomial logistic regression model was used to assess factors associated with a TB diagnosis, which was statistically significant with moderate explanatory power (χ²(6) = 15.7, p = 0.015, with a pseudo R² of 0.269). Significant predictors included age, history of TB in the past one to two years, and tobacco/alcohol use. Each additional year of age increased the odds of TB diagnosis by 7.27% (OR = 1.0727, p = 0.023). A recent history of TB strongly increased the likelihood of diagnosis (OR = 620.53, p < 0.001). Interestingly, tobacco and alcohol use were associated with significantly lower odds of TB diagnosis (OR = 0.0254, p = 0.0254). Other variables, such as the number of symptoms and history of diabetes, did not show significant associations with TB diagnosis (Table 4).

**Table 4:** Multivariate logistic regression analysis of predictors associated with TB diagnosis among individuals ^*^p < 0.05; ^**^p < 0.001; NS: not significant Multivariate logistic regression analysis was used The intercept indicates baseline log odds when all predictors are set to zero β: regression coefficient; SE: standard error; Z: Z-statistic; OR: odds ratio; TB: tuberculosis

Predictor	Estimate	SE	Z	p value	OR
Intercept	-23.5487	48,196.1428	-4.89 × 10^-4^	1^NS^	5.93 × 10^-11^
Age of family member (in years)	0.0702	0.0308	2.27574	0.023^*^	1.0727
Number of symptoms	-14.1561	3,219.582	-0.00440	0.996^NS^	7.11 × 10^-7^
History of TB in the last one to two years (yes)	6.4306	1.5709	4.0934	<0.001^**^	620.5271
History of diabetes (yes)	-17.1033	15,665.6811	-0.0011	0.999^NS^	3.73 × 10^-8^
Tobacco/alcohol use (yes)	0.0646	0.0360	7.608	0.0254^**^	0.0254

## Discussion

Our study found that symptoms such as appetite loss, weight loss, and general weakness were closely linked with individuals opting for TB testing. Among all the variables examined, a history of TB within the past one to two years emerged as one of the most significant predictors, both for deciding to get tested and for receiving a diagnosis. Interestingly, while almost one-third of individuals who reported appetite loss underwent testing, only four were confirmed to have TB. This gap between reported symptoms and actual diagnoses reflects a common limitation in symptom-based screening approaches.

Similar trends have been observed in national program evaluations. Between 2018 and 2020, India's ACF efforts showed considerable variation in quality and effectiveness across different states. During this time, improvements in testing methods, including the use of CBNAAT and chest radiography, helped reduce the number of people needed to screen to find one case. The national median number fell from 2080 in 2018 to 906 in 2020 [8]. In comparison, our study identified one case per 552 individuals screened, suggesting better efficiency. However, these findings must be interpreted with caution, as our data were drawn from a relatively small and specific population group, students and their household contacts in a tribal district.

Other community-based interventions have also used schools as entry points. For example, in 2021, a student-led TB screening initiative in Puducherry reached over 19,000 households but identified only three cases. While the diagnostic yield was modest, the model demonstrated operational feasibility and offered educational value for students [9]. Our study, though smaller in scale, reported a similar outcome, reinforcing the practical application of school-based ACF.

Despite the benefits, our approach did not prioritize individuals based on vulnerability, such as known exposure, existing health conditions, or socioeconomic status. This may have limited the overall diagnostic effectiveness. Previous global studies have emphasized that ACF can fall short, especially among marginalized populations, unless the strategy is adapted to the local context. Common barriers include social stigma, mistrust, and gaps in infrastructure [10]. While our study was conducted in a tribal setting, we did not categorize targeted risk factors, which may have reduced the potential impact of the intervention.

Some researchers have proposed that for ACF to work effectively, it must be well integrated into local health systems and supported by responsive infrastructure and learning tools. Although our study included data linkage with the Ni-Kshay surveillance platform, it did not involve deeper engagement with existing health service structures. Moreover, while we did not observe any negative consequences such as false positives or community resistance, other studies have raised concerns about possible harms when ACF programs are not implemented in a culturally sensitive manner [11,12].

Evidence continues to show that TB screening is most effective when focused on high-risk groups. A systematic review and meta-analysis by Garg et al. highlighted that groups such as people living with HIV, individuals with diabetes, known TB contacts, and members of tribal or rural communities have significantly lower numbers needed to screen to detect a case [4]. Our study setting, located within a tribal district, reflects some of these characteristics; however, without formally identifying high-risk households, our results may not fully reflect the efficiency possible in better targeted programs.

A national quality assessment conducted in 2023 reported that although nearly all districts in India carried out ACF activities, none met all the performance benchmarks set by the NTEP [13]. The average number of people who needed to be screened to find one case remained high, exceeding 2,800 in many areas. Compared with these figures, our study’s yield appears more promising. However, this could be influenced by factors such as sample characteristics and the structured involvement of students, rather than being a reflection of overall program quality.

Systematic reviews have shown that the highest yields from ACF come from programs conducted in settings such as HIV clinics, refugee camps, or health facilities [14]. Our school-based approach is not directly comparable to these high-risk environments and lacks formal involvement of CHWs or clinical staff, who have been identified in many studies as crucial facilitators of effective outreach [15]. Instead, we relied on students to engage with households, which, while innovative, may have affected the depth and accuracy of symptom reporting.

Even so, student-led models within tribal regions may still play an important role. Previous research has shown that consistent and community-focused case finding can significantly reduce TB prevalence. One study documented a decline in prevalence from 1,357 to 752 per 100,000 following ACF efforts in tribal areas [6]. Although our study did not aim to measure prevalence, the referral of symptomatic individuals for CBNAAT-based testing suggests that school-based interventions could make a meaningful contribution to TB control strategies.

Furthermore, patients diagnosed through ACF have been shown to have 1.4 times higher odds of completing treatment successfully compared to those diagnosed through passive surveillance [16]. This supports the case for investing in proactive detection efforts, even when the yield of cases seems modest. Finally, feedback from NTEP staff has highlighted several enabling factors for effective ACF, including dedicated budgets, access to diagnostic tools, and integration with digital platforms such as Ni-Kshay. However, ongoing challenges such as inadequate quality control and a generalized rather than targeted screening approach continue to limit success [17]. These constraints were also evident in our study, particularly due to the absence of a structured risk-based screening framework.

Nonconventional ACF approaches have proven effective in improving TB detection across various low-resource and hard-to-reach settings. In Nepal, CHWs implemented household and OPD-based ACF strategies, which led to a 13% increase in TB notifications and the identification of 1,193 additional cases [18]. In Bangladesh, peer-led screening among sexual minority populations significantly improved screening uptake and referral rates [19]. Similarly, a CHW-led door-to-door screening initiative in urban Indonesia demonstrated high community acceptance and operational feasibility, despite identifying no active cases in the pilot phase [20]. These models highlight the broader relevance of engaging nontraditional stakeholders in TB elimination strategies, especially in underserved regions.

Limitations

The cross-sectional design limits the ability to establish causal relationships between symptoms and the diagnosis of TB. The reliance on self-reported symptoms and voluntary participation may have introduced selection bias. Additionally, the study does not account for potential differences in the effectiveness of the screening process across various schools. The small number of TB diagnoses (four cases) may not fully represent the broader TB burden, limiting the generalizability of the findings. The lack of follow-up data means the long-term effectiveness of school-based ACF in sustaining TB diagnosis and treatment adherence remains unclear. Furthermore, cultural and social factors that could influence symptom reporting and testing uptake were not explored in-depth.

## Conclusions

Our findings suggest that involving school students in TB screening efforts within tribal communities is both practical and meaningful. By acting as health advocates, students were able to help identify people showing symptoms in their own households, encouraging timely medical attention. While only a few TB cases were ultimately diagnosed, the clear link between symptoms and testing highlights the potential of this approach. Considering its low cost and compatibility with national TB elimination efforts, this model could be worth expanding, though larger studies and longer-term follow-up are needed to understand its full impact.
